# Impact of Conventional Pasteurization, High Temperature Short Time, Ultra-High Temperature, and Storage Time on Physicochemical Characteristics, Bioactive Compounds, Antioxidant Activity, and Microbiological Quality of Fruit Nectars

**DOI:** 10.3390/foods13233963

**Published:** 2024-12-08

**Authors:** Natalia Polak, Stanisław Kalisz, Elżbieta Hać-Szymańczuk, Bartosz Kruszewski

**Affiliations:** 1 Department of Food Technology and Assessment, Institute of Food Sciences, Warsaw University of Life Sciences—SGGW, 02-776 Warsaw, Poland; natalia_polak@sggw.edu.pl; 2 Department of Biotechnology and Food Microbiology, Institute of Food Sciences, Warsaw University of Life Sciences—SGGW, 02-776 Warsaw, Poland; elzbieta_hac-szymanczuk@sggw.edu.pl

**Keywords:** thermal processing, food preservation, anthocyanins, vitamin C, strawberry, blackcurrant, chokeberry, microbiological safety, radical scavenging activity

## Abstract

Berries are a valuable source of numerous bioactive compounds, and they have an interesting organoleptic profile. Unfortunately, their low storage life determines the need for their preservation. Among the various methods used in this regard, it was decided to use the High Temperature Short Time (HTST) (90 °C/15 s) and Ultra-High Temperature (UHT) (130 °C/5 s) methods to preserve the produced fruit nectar blends (strawberry–blackcurrant and strawberry–chokeberry). For comparison, the nectars were also preserved using conventional pasteurization (90 °C/10 min). Physicochemical, chromatographic, and microbiological determinations were carried out in the tested nectars before and immediately after processing, as well as after 1, 2, 3, 4, and 6 months of refrigerated storage. All methods allowed for the significant inactivation of selected microbial groups. Non-significant changes were observed as a result of HTST and UHT processing in the context of pH, TSS, and titratable acidity. Varied major changes occurred in the content of bioactive components (TPC—decrease or increase by 2–4%, TAC—decrease by 3–20%, vitamin C—decrease by 15–78%), antioxidant activity (decrease or increase by 3–9%), and nephelometric turbidity (decrease or increase by 11–65%). Both nectars showed better quality and nutritional value after the HTST and UHT processes compared to treatment with classic pasteurization. Storage affected the degradation of bioactive compounds, reduced antioxidant activity, increased turbidity, and caused the brightening of samples together with reducing redness and yellowness. Considering the results obtained, it is reasonable to recommend the use of the HTST and UHT methods in industrial conditions for the preservation of liquid fruit and vegetable products such as juices, nectars, and beverages.

## 1. Introduction

Strawberry, chokeberry, and blackcurrant are very popular fruits in food processing. They are valued for both their sensory properties and their richness in bioactive compounds, which is why they are used in the production of jams, juices, and nectars.

Among the valuable components of strawberry (*Fragaria × ananassa* Duchesne) are organic acids (mainly citric and malic), mineral compounds (Ca, Fe, Mg, P, K, Na, Zn, Cu, Mn, and Se), vitamins (mainly ascorbic acid, but also vitamin A, E, K, biotin, thiamine, riboflavin, niacin, pantothenic acid, pyridoxine, and folic acid), and phenolic compounds [1]. A study covering 90 different varieties of strawberries grown in Poland confirmed a high content of phenolic compounds at levels ranging from 5.52 to 224.73 mg per 100 g fresh weight, while anthocyanins ranged from 0.31 to 92.59 in 100 g of fresh weight [2]. The rich composition of bioactive substances determines anti-cancer, anti-inflammatory, anti-diabetic, antioxidant, and also obesity-preventing or cardiovascular-protective properties [3].

Black chokeberry (*Aronia melanocarpa* (Michx.) Elliott) is also a valuable source of multiple bioactive compounds: phenolic compounds (anthocyanins, monomeric flavan-3-ols, phenolic acids, flavanones, and flavonols), vitamins (C, E, K, B1, B2, B3, B5, B6, and folic acid), and minerals (Mg, K, Ca, Fe, I, Mo, and Mn) [4]. Black chokeberry fruits contain 2000–8000 mg of polyphenols in 100 g of dry weight, of which 500 mg are anthocyanins [5,6]. Consumption of chokeberry can prevent cardiovascular diseases, control blood pressure, and favorably affect the course of metabolic and immune diseases. In addition, it protects the skin from UV radiation and reduces the negative effects of anti-cancer drugs, but it also exhibits anti-cancer effects itself. It can potentially inhibit the development of leukemia, breast cancer, and intestinal cancer. Black chokeberry also exhibits antimicrobial and antiviral properties, and is therefore used to treat certain subtypes of influenza viruses [7].

Blackcurrant (*Ribes nigrum* L.) is a fruit of which Poland was the world’s leading producer in the year 2023 [8]. Blackcurrant fruits are characterized by high vitamin C content of 50–342 mg/100 g of fruit [9,10]. In addition, they contain organic acids (citric, malic, maleic, and oxalic) and phenolic compounds (anthocyanins, phenolic acids, flavonols and flavan-3-ols, and procyanidin polymers) [11]. The content of phenolic compounds, including anthocyanins, in blackcurrant fruits varies over a wide range and is 498–1342 mg/100 g and 128–411 mg/100 g, respectively [12]. The peel of this fruit is particularly rich in anthocyanins [13]. Blackcurrant, thanks to its high content of bioactive compounds, exhibits antimicrobial, anti-inflammatory, antioxidant, and antihyperglycemic effects, strengthens the immune system, and helps collagen biosynthesis and the production of certain peptide hormones [9,10].

Berries have a low storage life, and are therefore commonly processed into juices, nectars, drinks, jams, preserves, or other products. Berries usually have a pH < 4.5, so they can be preserved using pasteurization. It typically uses temperatures of 80–90 °C for several minutes [14]. Despite destroying pathogenic or spoilage-causing microorganisms in food, such as, for example, *Escherichia coli*, *Staphylococcus aureus*, *Listeria monocytogenes*, and *Coxiella burneti* [15], and thus guaranteeing product safety, traditional pasteurization has a number of adverse effects. It significantly affects numerous quality characteristics of the final product—often negatively [16]—and thus can determine a decrease in desirability among consumers. In addition, it can lead to the degradation of monomeric anthocyanins, polymerization of anthocyanins, and browning of red fruit products [17]. Various thermolabile vitamins, such as ascorbic acid and riboflavin, are degraded during pasteurization. The availability of some essential amino acids has also been found to be reduced [15]. In this situation, new methods and parameters of preservation are being sought that could ensure better physicochemical and organoleptic quality of the final product while ensuring adequate shelf life. In the fruit and vegetable industry, investigations are ongoing regarding the use of innovative modern technologies like ohmic heating, microwave heating, high hydrostatic pressure, high-pressure homogenization, pulsed electric fields, high-intensity pulsed electric fields, ultrasound, supercritical carbon dioxide processing, UV light, pulsed light, oscillating magnetic fields, hydrodynamic cavitation, and micro- and ultrafiltration [18,19,20,21,22,23]. HTST (high-temperature-short time) pasteurization and UHT (ultra-high temperature) sterilization seem to be promising, thanks to their high efficiency and the possibility of testing different parameters, and they are being used more often in the food industry. The first method uses temperatures of up to 100 °C and times of a dozen to several tens of seconds, while the second uses temperatures above 100 °C and a time of several seconds. Based on our review of the literature [14] and consultations with beverage production facilities, the most commonly used parameters for HTST are 71.1–100 °C and 2–90 s, while those for UHT are 110–138 °C and 2–8 s.

The HTST and UHT methods are effective with properly selected parameters for the food matrix in inactivating microorganisms [24,25,26,27]. Lee et al. [28] demonstrated, using three different apple juices, that the color of HTST (72 °C/15 min) samples mostly changed to a similar or lesser degree than those preserved by pulsed electric field and high hydrostatic pressures compared to raw samples. For apple juice of the Jazz variety, ∆E* values were 8.34, 7.01, and 8.75, respectively, for the mentioned methods. Smaller changes (∆E* 0.14) in the color of mulberry juice due to the parameters of 110 °C/8.6 s, in comparison to raw samples, were presented by Zou et al. [29]. In cases involving the preservation of bioactive compounds, the HTST and UHT methods can be characterized by high efficiency. Amaro et al. [30], in a modeled study, indicated that processing at 70 °C, 80 °C, 90 °C, and 100 °C for 10 s resulted in vitamin C retention rates in orange juice of 0.92, 0.87, 0.85, and 0.82 compared to non-preserved juice. Heating can affect various phenolic compounds in different ways. Bonilla et al. [31] subjected a blackberry–soy–flaxseed-based beverage with three different ingredient formulations to rapid pasteurization at 71.1 °C/3 s and traditional hot-filling at 87 °C. Each beverage subjected to HTST retained more cyanidin-3-*O*-glucoside and cyanidin-3-*O*-malonyl-glucoside and total phenolic compounds than the hot-filled samples. In the study of Wang et al. [32], UHT preservation of tomato juice at 110 °C/8.6 s determined a significant decrease in the content of quercetin, while no significant change occurred in caffeic and chlorogenic acids. Furthermore, the UHT samples were not significantly different from fresh and HHP samples in regard to their phenolic acid contents. We present a more detailed discussion and comparison of scientific reports about the HTST and UHT methods’ influence on enzymes, microbial inactivation, and retention of bioactive compounds in our review paper [14].

In the literature, little attention has been paid to the impact of HTST and UHT processing on the quality of fruit and vegetable nectars. However, there is a great need for such information in food industry, particularly when berries are processed (due to their low shelf life, as well as the unacceptable sensory characteristics of some varieties). Furthermore, the HTST and UHT methods’ utilization data have high importance for the food industry due to the use of innovative compact flow pasteurizers. Therefore, the aim of this study was to determine the effect of these continuous-flow preservation methods on the physicochemical and microbiological quality of two types of nectars. An attempt was also made to determine the importance of the food matrix in terms of changes in the quality of the preserved samples. The scope of this study also includes storage studies covering up to 6 months of refrigerated storage.

## 2. Materials and Methods

### 2.1. Materials

The black chokeberry (*Aronia melanocarpa* (Michx.) Elliott), strawberry (*Fragaria × ananassa* Duchesne), and blackcurrant (*Ribes nigrum* L.) fruits samples were obtained between July and September 2023 from one of the plantations located close to Czerwińsk nad Wisłą city (coordinates 52.39858, 20.30970) in central Poland. The fruits were picked up at harvest maturity, as declared by the growers, an then were stripped of inedible parts and immediately frozen until production of the nectar occurred.

### 2.2. Methods

#### 2.2.1. Preparation, Preservation, and Storage of Nectars

Direct juices were pressed using a semi-industrial hydraulic press HPL14 (BUCHER Unipectin AG, Niederweningen, Switzerland) from the gathered raw materials. Strawberry, currant, and chokeberry nectars contained 40%, 25%, and 25% of juice, respectively, and were adjusted to 1% acidity in accordance with European Union law [33]. Mixed nectars were then prepared with equal proportions of single-ingredient nectars (50/50): (1) strawberry–blackcurrant nectar (SB) and (2) strawberry–chokeberry nectar (SC).

Next, obtained nectars were preserved using traditional pasteurization (PT), HTST (High Temperature Short Time), and UHT (Ultra-High Temperature) methods. Based on a review of the literature [14] and consultations with manufacturers, we chose preservation parameters of 90 °C/15 s for the HTST and 130 °C/5 s for the UHT method. A tubular pasteurizer FT74XA HTST/UHT System (Armfield, Ringwood, UK) with adjusted flow rate was used with the above preservation parameters. The bottling was performed in a laminar chamber (FT83 Sterile Filling System, Armfield, Ringwood, UK) with HEPA filters into 80 mL glass jars. PT samples were closed into glass jars with the same volume and then heated 10 min at 90 °C in a water bath. The next step involved cooling to 20 °C.

Nectars were subjected to physicochemical, chromatographic, and microbiological analyses before preservation, immediately after preservation, and during 6-month refrigerated storage (after 1, 2, 3, 4, and 6 months). The control sample was raw nectar before preservation.

#### 2.2.2. Physicochemical Parameters Measurement

Total soluble solids (TSS) were determined using a digital refractometer (Refracto 30PX, Mettler Toledo, Columbus, OH, USA) made in accordance with the operating instructions. Results were expressed in °Brix.

Active acidity (pH) was determined using a pH-meter (CPI-601 Elmetron, Zabrze, Poland) following the operating instructions. Titratable acidity (TA) was determined using a compact titrator (SI Analytics Titrator TitroLine^®^ 5000, Xylem Analytics Germany Sales GmbH & Co. KG., Weilheim in Oberbayern, Germany). The samples of nectars were titrated with 0.1 M sodium hydroxide solution until a pH of 8.1 was reached. The results were expressed as g of citric acid per 100 mL of nectar.

Nephelometric turbidity (NT) was determined using a 2100Q portable turbidimeter (Hach Lange GmbH, Germany) following the operating instructions. The results were expressed in nephelometric turbidity units (°NTU) at 20 °C. The device was calibrated against formazin turbidity standards.

#### 2.2.3. Color Parameters Measurement

Before measurements, nectars were centrifuged at 14 000 rpm/min for 10 min in a laboratory centrifuge (MPW-350R, MPW MED. INSTRUMENTS, Warsaw, Poland). Color parameters in the CIE L*, a*, b* system were determined using a CM-3600d colorimeter (Konica Minolta, Osaka, Japan) in the mode of transmitting light. Like in previous studies, the D65 illuminant, an observer 10°, and the port size set to 25.4 mm were used [20]. Measurements were conducted with a glass cuvette with a layer thickness of 10 mm. Moreover, the total color difference between (1) treated and non-treated samples and between (2) samples after heat treatment at 0 days and after 6 months of storage was calculated using Equation (1).

(1) $${∆E}^{*}=\sqrt{{{(∆L}^{*})}^{2}+{{(∆a}^{*})}^{2}+{{(∆b}^{*})}^{2}}$$

#### 2.2.4. Vitamin C Content Determination

Vitamin C content in samples was determined using high-pressure liquid chromatography with a diode array detector (HPLC-PDA, Shimadzu, Japan). The nectars were filtered through 0.45 µm PTFE syringe filters into glass vials. The analysis was conducted in an isocratic flow on an Onyx Monolithic C18, 100 × 4.6 mm column (Phenomenex), at 25°C and with a flow rate of 1 mL/min. The 0.1% H3PO4 was the mobile phase [20]. The results were recorded at a wavelength λ = 254 nm. Identification was conducted on the basis of a standard chromatogram. The results were expressed as mg per 100 mL of nectar.

#### 2.2.5. Anthocyanin Content Determination

Anthocyanin content in nectar samples was determined using high-pressure liquid chromatography with a diode array detector (HPLC-PDA, Shimadzu, Japan) in accordance with the methodology presented by Goiffon et al. [34]. The nectars were filtered through 0.45 µm PTFE syringe filters into glass vials. The analysis was conducted in an isocratic flow on a Luna 5 μm C18(2) 250 × 4.6 mm column (Phenomenex) at 25 °C and with a flow rate of 1 mL/min. A mixture of water, acetonitrile, and formic acid in the ratio 81:9:10 (*v*/*v*/*v*) was used in the mobile phase. The results of anthocyanins were recorded at a wavelength λ = 520 nm. Monomers of the anthocyanins were identified by comparing their retention times based on a self-made library of selected anthocyanin standards. Standards were bought from Extrasynthese (Genay, France) and Biosynth Ltd. (London, UK). The content of individual anthocyanins, as well as the total amount of anthocyanins (TAC), were expressed as mg of cyanidin-3-O-glucoside per 100 mL of nectar.

#### 2.2.6. Total Phenolic Content Determination

Total phenolic content (TPC) in nectar samples was determined using Folin–Ciocalteau’s reagent method in accordance with the methodology presented by Gao et al. [35]. Before measurements, nectars were centrifuged at 14,000 rpm/min for 10 min in a laboratory centrifuge (MPW-350R, MPW MED. INSTRUMENTS, Poland). Then, the proper dilutions of the supernatants were made so that the absorbance fell within the determined standard curve. Then, 0.1 mL of sample solution was mixed with 0.2 mL of Folin–Ciocalteau’s reagent, 1 mL of 15% sodium carbonate, and 2 mL of redistilled water. After 1 h of mixture incubation in the dark at 25 °C, absorbance was measured using a UV-VIS spectrophotometer (UV1650PC, Schimadzu, Japan) at a wavelength λ = 765 nm against the mixed reagents. TPC was expressed as mg of gallic acid equivalents (GAE) per 100 mL of nectar.

#### 2.2.7. Antioxidant Activity Determination

Antioxidant activity (AA) was determined using DPPH (2,2-diphenyl-1-picrylhydrazyl) radicals, in accordance with the methodology presented by Yen and Chen [36], with some modifications. Before measurements, nectars were centrifuged at 14,000 rpm/min for 10 min in a laboratory centrifuge (MPW-350R, MPW MED. INSTRUMENTS, Warsaw, Poland). Dilutions of supernatants were made so that the absorbance fell within the determined standard curve. Then, 1 mL of sample solution was mixed with 3 mL of methanol and 1 mL of DPPH radicals. After 10 min, the absorbance was measured using a UV-VIS spectrophotometer (UV1650PC, Schimadzu, Japan) at a wavelength λ = 517 nm.

The antioxidant activity of samples was also determined with ABTS (2,2′-azinobis-(3-ethylbenzothiazoline-6-sulfonic acid)) radicals, in accordance with the methodology presented by Re et al. [37]. Before measurements, nectars were centrifuged as described above. Dilutions of supernatants were made so that the absorbance fell within the determined standard curve. Then, 40 μL of sample solution was mixed with 4 mL of ABTS radicals. After 6 min, the absorbance was measured using a UV-VIS spectrophotometer (UV1650PC, Schimadzu, Japan) at a wavelength λ = 734 nm.

The results for both DPPH and ABTS tests were calculated using Equation (2) and are expressed as RSA (radical scavenging activity %).

(2) $$RSA\left[\%\right]=\frac{Ac-As}{Ac}\times 100\%$$

where Ac is the absorbance at 517 or 734 nm of the control sample and As is the absorbance at 517 or 734 nm of the test sample.

#### 2.2.8. Microbial Analysis

The samples were subjected to microbiological analyses to determine the content of mesophilic aerobic microorganisms (MAMs), lactic acid bacteria (LAB), endospore-forming bacteria (EFB), acetic acid bacteria (AAB), *Enterobacteriaceae* (EB), and yeast and mold (Y&M). The bacterial counts were expressed as colony-forming units (CFU) per 1 mL of nectar.

MAMs were determined based on the requirements of the ISO 4833-1:2013 [38] standard using PCA medium (BTL, Łódź, Poland).

LAB were determined based on the requirements of the ISO 15214:1998 [39] standard in MRS medium (Bio-Rad Laboratories, Inc., Hercules, CA, USA).

EFB and AAB were determined based on the protocol published by Kim et al. [40] using PCA and GYC agar medium (Merck KGaA, Darmstadt, Germany), respectively.

EB was determined based on the requirements of the ISO 21528-1:2017 standard [41] using VRBG agar medium (BTL, Łódź, Poland).

Y&M was determined based on the requirements of the ISO 21527-1:2008 [42] in DRBC medium (BTL, Łódź, Poland).

#### 2.2.9. Statistics

All data are presented as a mean with standard deviation for three replications of each measurement carried out for each of two independent repetitions of thermal treatment experiments. Mean results and standard deviations were calculated using Microsoft Excel 2013. Statistical analysis was performed in the Statistica 13.3 program (TIBCO Software Inc., Carlsbad, CA, USA) using analysis of variance (ANOVA) and Tukey’s HSD post hoc test in order to check the significance of the differences between preservation methods, storage time, and nectar variants, with the significance level α = 95%. Moreover, Pearson’s correlation test was used in order to check correlations between the content of bioactive compounds, color parameters, and antioxidant capacity.

## 3. Results and Discussion

### 3.1. pH, Titratable Acidity (TA), Total Soluble Solids (TSS), and Nephelometric Turbidity (NT)e

Total soluble solids (TSS) and titratable acidity (TA) are the parameters that determine the organoleptic characteristics of fruit and vegetable products. pH determines the choice of food preservation method. A change in these parameters can be associated with microbiological growth, and is also an indicator for assessing product safety. Due to the small changes in pH, TA, and TSS values, only the results for the raw samples immediately after preservation, as well as after the sixth month of storage, are shown (Table 1). The results show that HTST and UHT nectars do not significantly alter the pH, TSS, or TA of the nectars tested immediately after the process or during refrigerated storage. Therefore, the HTST and UHT methods allow us to maintain similar levels of the basic physicochemical parameters of produced nectars in comparison to both raw and PT samples. The literature data confirms the occurrence of the above trend [14].

Turbidity (NT) measurements allow us to determine the clarity of juice and the concentration of suspended particles and colloids present in liquid samples [43]. Visual assessments of turbidity in liquid products can be an important factor in consumers’ sensory evaluation [44], so it is important to monitor product turbidity after preservation as well as during storage. Raw SB nectar had five times higher turbidity than SC (Table 2). Significant changes in NT were observed after preservation of the tested nectars. For both UHT-treated nectars, turbidity was significantly lower than in the raw samples. There was a 16%, 18%, and 65% NT reduction in SC nectar immediately after preservation using the PT, HTST, and UHT methods, respectively. During 6 months of storage, the UHT-treated sample remained at 73°NTU, while for the PT and HTST methods, the NT increased by 9% and 40%, respectively. A different relationship was observed in the SB nectar. The PT and UHT methods caused a decrease in NT of 11% and 36% immediately after processing, while the HTST method caused an increase of 11%. During the entire storage period, there was an increase in NT by 52%, 6%, and 38% in SB nectar samples preserved using the PT, HTST, and UHT methods, respectively. In the case of SB nectar, UHT-treated samples had significantly lower turbidity in each month of storage than those treated using the other methods. For SC nectar, this observation was valid for only 4 months.

Turbidity is related to the presence of various compounds like proteins, fats, pectins, cellulose compounds, and their complexes with other substances [20]. The varying effects of the same preservation parameters on the NT of different nectars may be related to their microstructure. Turbidity changes may be associated with various physicochemical processes, such as precipitation of larger particles, polymerization of phenolic compounds and proteins, or non-enzymatic depolymerization of pectins caused by elevated temperatures [43,44,45]. It should be noted that the UHT method in both nectars is a far more favorable method regarding turbidity in comparison to the PT method. This is due to the greater reduction in NT immediately after the UHT process as well as at the end of the storage period.

Also, the literature data indicate the diverse effect of the chosen methods on the turbidity of juices and beverages. A similar magnitude of NT change compared to HTST-treated SB nectar was observed by Bonilla et al. [31] in three different formulations of blackberry–soy–flaxseed beverages (an increase of 6.4–15.5% due to heating at 71.1 °C/3 s). About one-third less of a change was observed in peach juice preserved at 72 °C/15 s [46] compared to SC nectar processed using HTST. Liu et al. [47] showed only a 2% decrease in the turbidity of cucumber juice following treatment at 110 °C/8.6 s, which is a much smaller change than in our study. Tian et al. [43] showed a 110% increase in turbidity in cloudy pomegranate juice following heating at 110°C for 8.6 s.

### 3.2. Nectar Color Changes Due to Preservation Method and Storage Time

**Figure 1:**
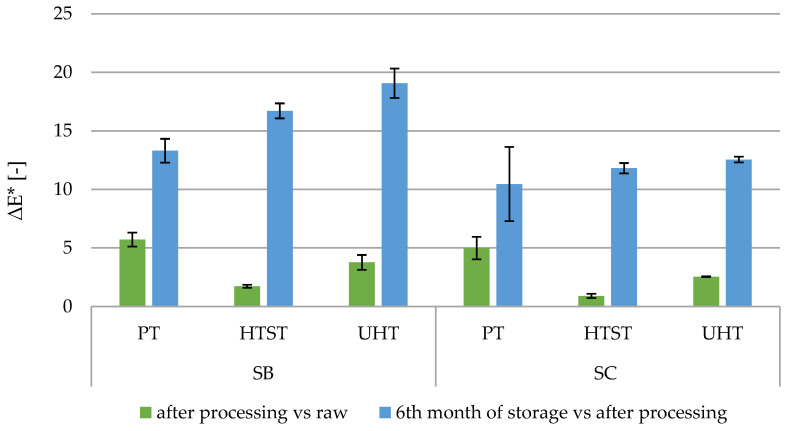
Total color difference (∆E*) between nectar samples. PT—traditional pasteurization, HTST—high temperature short time, UHT—ultra-high temperature, SB—strawberry–blackcurrant nectar, SC—strawberry–chokeberry nectar.

The SC nectar, in comparison to SB, was characterized by a 34% higher level of brightness, a 23% lower level of redness, and a 41% lower level of yellowness. During storage in both nectars, the parameters a* and b* decreased significantly, while L* increased significantly. The largest color changes were observed when using the PT method, and the smallest changes were observed when using the HTST method (Table 3). The change in the value of the b* parameter was most responsible for the change in the overall color of the nectars. After 6 months of storage, the color changed to the greatest extent in samples after using the UHT method, and the least after using the PT method. Based on the information presented by Pérez-Magariño and González-Sanjosé [48] the human eye is able to perceive a color difference when ΔE* ≥ 1, but when observation occurs through glass, it can only distinguish color when ΔE* ≥ 5. ΔE immediately after the treatment of SB nectar was 5.71, 1.73, and 3.77 for the PT, HTST, and UHT methods, respectively, while for SC nectar, the values were 4.99, 0.90, and 2.54 (Figure 1). Comparing the samples which were sealed in glass jars, it can be concluded that a change of color was visible for an average observer only for the PT samples. Visible changes in color may discourage potential consumers from consuming the product, as they may connote a reduction in quality or even spoilage.

The pH determines the color of anthocyanin compounds; in an acidic environment, they are characterized by red coloration (reflected by a positive value of the parameter a* in ours nectars). Considering the method of preservation, a longer heating time during processing resulted in a greater effect on color change, probably related to the degree of degradation of anthocyanin pigments and the degree of induction of browning reactions. The use of a shorter heating time during the HTST and UHT methods preserved color similar to the raw samples. It was most likely related to the degree of degradation in anthocyanin pigments and the degree of induction of browning reactions, which are also dependent on the content of sugars, polyphenolic compounds, and vitamin C. Thermal transformations of the mentioned compounds can lead to the formation of colorful reaction products. pH, due to small changes, did not affect the color change of anthocyanin compounds, whose color depends on the environmental conditions. During storage, the color changes resulted mainly from the breakdown of color compounds.

In our study, thermal preservation caused a brightening of color and a reduction in the contribution of red and yellow colors. Khalil et al. [49] observed a brightening of carrot–orange juice and an increase in the values of the a* and b* parameters. In our study, the ΔE* was less than 4 for the UHT and HTST samples. The literature reveals a varying impact of the mentioned methods on this indicator. Tian et al. [43] obtained, in pomegranate juice, ΔE* = 5.19 and 6.62 for 85 °C/30 s and 110 °C/8.6 s conditions, respectively. These values were higher than in our study. However, there is agreement regarding the greater effect on color of the UHT method. An even more significant change in total color difference than in the mentioned studies was observed in lime juice ΔE* = 11.4 treated with 136°/4 s [50]. You et al. [51] compared the traditionally used parameters of 70 °C/10 min and 75 °C/10 min with a variant of 110 °C/8.6 s to preserve mulberry juice. Their ΔE* values, relative to the control sample, were 16.78, 29.83, and 8.04, respectively. Although these values are significantly higher than those obtained by us, they are consistent with our observation that traditional, prolonged pasteurization has a greater impact on color change than the HTST or UHT methods. On the other hand, reports of small color changes caused by the interaction of high temperature and short time can be found in the literature. For example, Bonilla et al. [31], using 71 °C/3 s observed ΔE* in a range of 1.7 to 2.6 depending on the formulation of blackberry–soy–flaxseed beverage, and Estrada-Beltrán et al. [26] calculated ΔE* = 1.54 in apple–raspberry juice using processing conditions of 85 °C/6 s.

### 3.3. Impact of Preservation Methods and Storage Time on Vitamin C Content

The SB nectar had a significantly higher vitamin C content (31.78 mg/100 mL) than SC (5.89 mg/100 mL) (Table 4). Each method significantly affected the degradation of this thermolabile component in both nectars. In SC nectar, the changes were significantly higher after preservation; we observed a decrease of 77%, 78%, and 68% for the PT, HTST, and UHT methods, respectively. In SB nectar, the decreases were 24%, 20%, and 15%, respectively. A significant downward trend in vitamin C content was also observed during storage. The greatest changes were observed after 1 month of storage (decreased of 48%, 76%, and 74% in SB and 54%, 46%, and 68% in SC nectar for the PT, HTST, and UHT methods, respectively). For further storage, the rate of degradation varied between nectars, as well as preservation methods, which may be due to chemical composition and different heat exposition. After 6 months, in comparison to the beginning of storage, 48%, 4%, and 3% (SB nectar) and 48%, 57%, and 37% (SC nectar) of vitamin C remained in the samples treated using the PT, HTST, and UHT methods, respectively.

The degradation of vitamin C over time is consistent with the literature data [16,52]. Degradation is mostly related to the effects of oxygen, causing the reversible oxidation of ascorbic acid to dehydroascorbic acid. Further dehydroascorbic acid irreversibly hydrolyzes to 2,3-diketogluconic acid or causes the formation of 3-hydroxy-2-pyron, 2-furoic acid, furfural, and others. The literature data indicate that, at temperatures above 100 °C, ascorbic acid degradation is more affected by oxygen, so it is important to remove oxygen from the sample as much as possible, including dissolved oxygen [45,53,54]. In our study, it was also associated with the methods’ parameters. However, other factors, such as pH, exposition to light, contact with the metal surfaces of machines, or the presence of enzymes, must be taken into account [45,55].

de Souza et al. [56] also indicate the differential behavior of vitamin C depending on the food matrix treated under the same conditions; the researchers observed a 92% decrease in lemonade and a 12% decrease in citrus juice due to HTST (75 °C/90 s). Also, Xu et al. [57] observed significant differences in decreased vitamin C between clear and cloudy selenium-enriched kiwifruit juice processed using the UHT method. Yuan et al. [58] compared the effect of different preservation conditions, 85 °C/30 s and 110 °C/8.6 s, of cloudy pomegranate juice and indicated, in contrary to our observations, that higher temperature, together with shorter time, directly causes greater vitamin C degradation. In our study, UHT processing allowed for the greatest retention of vitamin C in both nectars, but after the full length of storage, in these samples, we determined the least amount of this compound.

**Figure 2:**
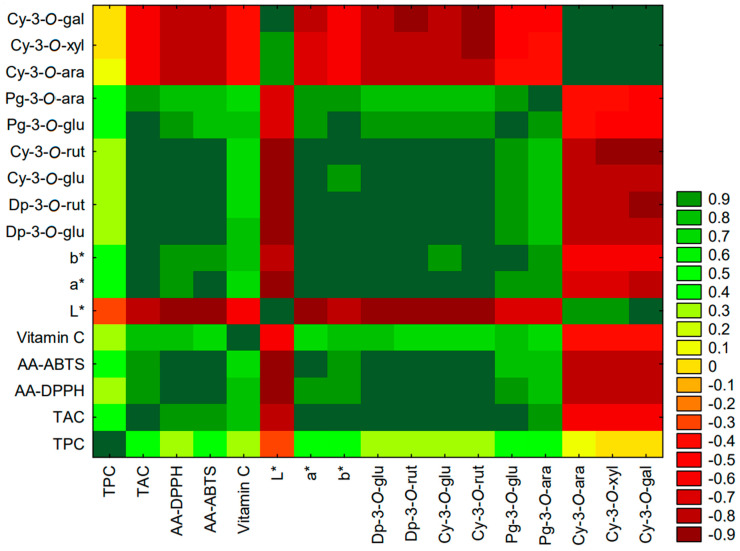
Heatmap of correlations between content of bioactive compounds (TAC, TPC, vitamin C content, individual anthocyanins), color parameters (L*, a*, b*), and antioxidant activity (AA), based on total results obtained for all samples.

The literature indicates that polyphenolic compounds can have a protective effect on vitamin C [53]. In the present study, there was a weak correlation (R = 0.26) between total polyphenol and vitamin C content, but a high correlation (R = 0.78) between the sum of anthocyanins and vitamin C content (Figure 2).

### 3.4. Impact of Preservation Methods and Storage Time on Anthocanin Content

**Figure 3:**
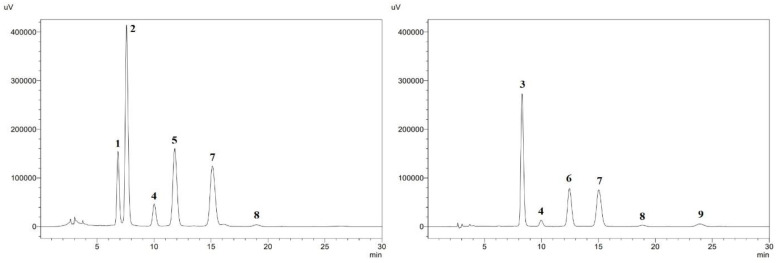
Chromatograms of anthocyanins present in raw nectars: strawberry–blackcurrant (SB) nectar on the left, and strawberry–chokeberry (SC) nectar on the right. Identified anthocyanins: 1—delphinidin–3–O–glucoside, 2—delphinidin–3–O–rutinoside, 3—cyanidin–3–O–galactoside, 4—cyanidin–3–O–glucoside, 5—cyanidin–3–O–rutinoside, 6—cyanidin–3–O–arabinoside, 7—pelargonidin–3–O–glucoside, 8—pelargonidin–3–O–arabinoside, 9—cyanidin–3–O–xyloside.

Based on the chromatographic analysis of the nectars, we identified nine anthocyanins in total. Chromatograms with identified individual anthocyanins are shown in Figure 3. In SB nectar, the delphinidin–3–O–rutinoside was present in the highest amount, accounting for 29% of the total anthocyanins (TAC) in the raw sample. In SC nectar, it was cyanidin–3–O–galactoside with the highest share of 15% of TAC in the raw sample.

A significantly higher total amount of anthocyanin was found in SB than in SC nectar (about 48% more) (Table 4). A statistically significant decrease in TAC was observed in both nectars after preservation using either method, as well as during storage. In the case of SB nectar, the highest amount of determined anthocyanin was retained in samples preserved using the HTST method: 97% after preservation and 52% after 6 months of storage. Also, for the SC nectar, the HTST method was the best; it preserved 96% of TAC after treatment and 58% at the end of storage. Looking at the results, it can be observed that anthocyanins were more stable in SB nectar. Considering the months of storage, it was shown that, in the tested nectars, the content of anthocyanin compounds was more stable during the initial storage period. The smallest changes occurred at the fourth month in both nectars. The biggest changes occurred in the last month, because we observed TAC degradation in SB and SC nectars in the range of 14–21%.

Considering individual anthocyanins, immediately after preservation in SB nectar, it was PT that had the least, while the UHT method had the greatest effect on the content of these compounds (Table A1). In the case of SC samples, it showed a more nuanced relationship. For example, the HTST method retained cyanidin-3-O-galactoside and pelargonidin-3-O-arabinoside the best, while UHT treatment reduced their content the most. The PT method maintained cyanidin-3-O-glucoside and cyanidin-3-O-arabinoside to the greatest extent, while UHT processing degraded them more intensely. After 6 months of storage in SB nectar, pelargonidin-3-O-glucoside and delphinidin-3-O-glucoside were degraded the most, while pelargonidin-3-O-arabinoside was degraded to the least extent among all anthocyanins. For SC nectar, cyanidin-3-O-arabinoside was retained the most, while pelargonidin-3-O-arabinoside content was highly diminished.

In our study, the HTST and UHT methods proved to be the better methods in terms of anthocyanin retention immediately after the process compared to traditional pasteurization (PT). Reducing the time of exposure to high temperature had a beneficial effect on the retention of these bioactive components. In the literature, equally low changes (decreases of 3–7%) due to HTST (90 °C/5 s) processing have been shown in juices from different pomegranate varieties [59]. In another study, UHT (110 °C/8.6 s) treatment of pomegranate juice showed a similar results to us: a 13% decrease in anthocyanin content [60]. Yuan et al. [58] also compared different parameters of thermal preservation, indicating, as we did, that HTST-type conditions (85°C/30 s) retained total anthocyanin content better than the UHT method (110 °C/8.6 s).

High correlations were observed between TAC and the parameters L* (R = −0.90), a* (R = 0.93), and b* (R = 0.99), much higher than with TPC (−0.31, 0.43, and 0.39, respectively). For individual anthocyanins and the L*, a*, and b* parameters, the correlation values ranged between 0.62 and 0.98 (Figure 2).

### 3.5. Total Phenolics Content (TPC)

About one third more TPC was determined in SC nectar than in SB (Table 4). Preservation of SB nectar reduced TPC slightly by 4%, 2%, and 4%, while in SC nectar, TPC slightly increased by 4%, 2%, and 3%, for the PT, HTST, and UHT methods, respectively. But all of these changes were statistically insignificant. Over 6 months of storage of SB nectar, only in HTST samples we have observed statistically significant decreases in TPC. But in SC nectar, after the same period of time, all of the variants significantly lowered TPC. During storage, the HTST method had the most degrading effect, while the PT method had the least. TPC changes in SB nectar were distributed proportionally similar in each month. In the case of SC nectar, the greatest changes occurred after the first (44–46%) and sixth months (28–30%).

In the case of our SB nectar, small changes in TPC were observed by de Souza et al. [56]: in lemonade and citrus juice, TPC decreased slightly by 4% and 8%, respectively, due to HTST (75 °C/90 s) processing. A decrease of 3% in TPC was shown by Mesta-Vicuña et al. [27] in red prickly pear juice due to preservation at 130 °C/3 s; this is similar to observations in our SC nectar after UHT treatment. Also, Liu et al. [52] and Xu et al. [61] indicated that there were no significantly statistical changes after preservation with 110°C/8.6 s in mango nectar or orange–pepper juice. Xu et al. [61] indicated that the UHT juice had 91% less TPC after 25 days of refrigerated storage, a far greater change than after one month of storage in our SB (2% decrease) or SC (45% decrease) nectars treated with 130°C/5 sec. Liu et al. [52] observed a 17% decrease in TPC in mango nectars processed with 110°C/8.6 s and stored 16 weeks at 4°C. In comparison, after four months of cold storage of UHT samples, we determined 6% and 41% decreases in SB and SC nectars, respectively.

Phenolic content depends on factors such as temperature, time, heat dose, presence of oxygen and light, pH, the presence of co-pigments, proteins, sugars and metal ions, chemical structure, and the concentration of anthocyanin compounds. Their transformation is also associated with numerous processes carried out on fruits and vegetables, such as tissue disintegration, activity of native and added enzymes, oxidation during pressing or mixing, and others [14,45]. Therefore, depending on the food matrix, which determines physicochemical transformations, a decrease as well as an increase in the content of polyphenolic compounds can occur with the same preservation parameters.

### 3.6. Influence of Preservation Methods and Storage Time on Antioxidant Activity

The antioxidant activity (AA) of SB nectar measured with DPPH and ABTS radicals did not change significantly after preservation, while a significant decrease was observed in each variant after 6 months of storage. No significant changes in DPPH values were observed in SC nectar after preservation, as well as during storage. In this nectar, different relationships were observed after measurements with ABTS: (1) antioxidant activity increased significantly after thermal processing, (2) no significant changes were observed at the end of the storage period in samples after PT, and (3) a significant decrease was observed at the end of the storage period in samples after using the UHT and HTST methods. A greater level of change during storage occurred in SB nectar in both assays; after 6 months, there was a decrease in antioxidant activity after PT, HTST, and UHT treatment of 30%, 25%, and 24% (DPPH) and 19%, 21%, and 18% (ABTS), respectively.

**Figure 4:**
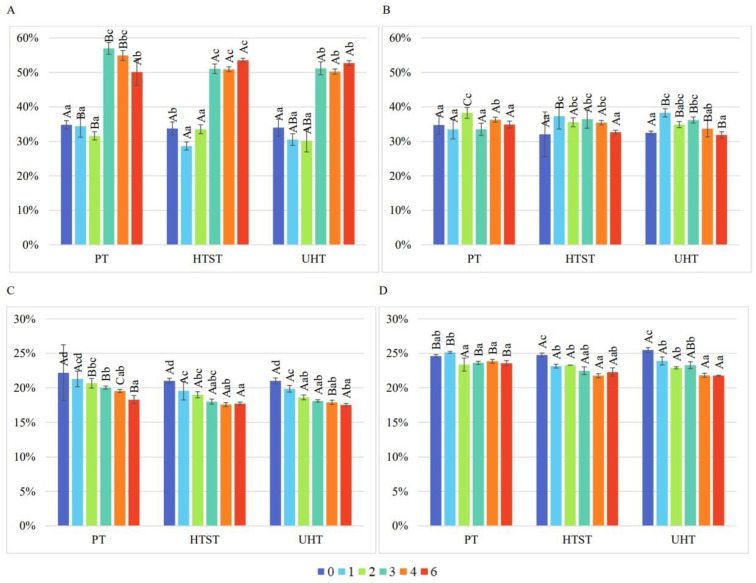
DPPH radical scavenging activity [%] of strawberry–blackcurrant nectar (**A**) and straw-berry-chokeberry nectar (**B**); ABTS radical scavenging activity [%] of strawberry–blackcurrant nectar (**C**) and strawberry–chokeberry nectar (**D**). HTST—high temperature short time, PT—traditional pasteurization, UHT—ultra-high temperature. Values marked with different capital letters are significantly different (*p* < 0.05) and concern changes between preservation methods in SB or SC nectars in specific weeks of storage. Values marked with different small letters are significantly different (*p* < 0.05) and concern changes during storage in SB or SC nectars for specific preservation methods.

Figure 4 shows the DPPH and ABTS radical scavenging activity of nectars as a function of preservation method and storage time. In the case of SB nectar, the apparent trend of increasing activity during storage measured with the DPPH assay and decreasing with the ABTS assay is associated with a decrease in antioxidant activity. In contrast, in the SC nectar, changes were smaller and more varied compared to when the DPPH assay was used.

Zou et al. [29] showed a non-significant change in AA using the DPPH method in mulberry juice treated with 110 °C/8.6 s, which is consistent with our observations for both nectars. Sweet potato nectar also showed no significant change in AA after UHT preservation [62]. The indicated examples are consistent with our observations for both nectars measured using DPPH; the lack of significant change was likely related to the absence of significant TPC transformations. Yildiz and Aadil [63] indicated an increase of 27% in AA in strawberry juice following treatment at 72 °C/15 s, which is far greater than in our SC nectar after HTST. Mesta-Vicuña et al. [27] observed a significant decrease in AA by almost 60% in red prickly pear juice after both 80 °C/30 s and 130 °C/3 s processing. Similar observations to our study were made by Khalil et al. [49]: immediately after preservation (72 °C/5–21 s), there was a decrease in AA in samples by 6–19%. For all of our samples, AA decreased during storage, which is in line with the results reported by other researchers [52,63].

Antioxidant activity was more strongly correlated with TAC (R = 0.88 for DPPH and R = 0.86 for ABTS) than with TPC (R = 0.24 and R = 0.31) (Figure 2). Also, a strong correlation was observed between antioxidant activity measured using the DPPH and ABTS assays and vitamin C content (R = 0.74 and R = 0.69, respectively). Considering the individual labeled anthocyanins, cyanidin–3–O–rutinoside was connected the most with the antioxidant activity of samples, and pelargonidin–3–O–arabinoside has the least linkage.

### 3.7. Microbiological Characteristics of Nectars

Mesophilic aerobic microorganisms (MAMs), lactic acid bacteria (LAB), acetic acid bacteria (AAB), and yeast and mold (Y&M) were detected in raw nectars in varying contents, which was related to the different nectars’ compositions (Table 5). Spore-forming bacteria (EFB) and *Enterobacteriaceae* (EB) were not identified in the raw samples. All analyzed samples had a pH below 4.5, which confirmed the applicability of temperatures below 100 °C in this study.

In the case of SC nectar, each preservation method caused a significant decrease in MAMs and AAB number, while in SB nectar, a significant change did not occur only for the HTST method. Complete inactivation immediately after preservation in both nectars was observed for LAB and Y&M. Heat causes changes in various parts of microorganism cells, such as the outer and inner membrane, peptidoglycan cell wall, enzymes, nucleoid, RNA, or ribosomes. As a result, some microorganisms can be inactivated, and as a result, their multiplication potential has been inhibited. Due to unfavorable environmental conditions (such as low pH and limited oxygen content, like in the case of our study), cells eventually undergo autolysis. Insufficiently damaged cells, under the right conditions, can undergo regeneration [64]. Therefore, for some of the analyzed groups of microorganisms in our study, their presence can be observed immediately after the process, at the first month of storage as well as at the sixth (Table 5). In the case of AAC, the low pH may promote cell regeneration, hence their possible appearance at the end of the storage period in the nectars studied; however, no significant relationship was noted between pH and titratable acidity and the presence of AAB in samples. The high shelf life (6 months) of both nectars treated using PT and continuous flow heat processing (UHT and HTST) is related not only to their low pH, but also to the content of antimicrobial bioactive components in the studied nectars.

Mesta-Vicuña et al. [27] observed that different thermal treatments (80 °C/30 s, and 130 °C/3 s) eliminated the microbial load (total coliforms, yeast and mold, mesophilic, and psychrophilic) after immediate treatment and during storage for 20 days in red prickly pear juice. In watermelon juice preserved at 110 °C/2 s, 120 °C/2 s, and 135 °C/2 s, it was observed that the survival rate of total flora count was below 0.01%, 0.1%, and 0.01%, respectively [24]. Also, numerous other studies indicate the effectiveness of the HTST and UHT methods in inactivating microorganisms [16,65,66].

In the case of SC nectar, it can be concluded that the methods used were not significantly different from each other and allowed for the significant inactivation of microorganisms, while in the case of SB nectar, only the samples subjected to HTST were not statistically significantly different from the raw sample in terms of MAMs and AAB. However, it should be strongly emphasized that the nectars produced meet the criterion for pasteurized juices, limiting the number of mesophilic microorganisms at 10^3^ – 10^4^. The effective elimination of the selected groups of microorganisms, as well as the absence of pathogens, confirms the validity of the use of traditional pasteurization (PT) and the HTST and UHT methods.

## 4. Conclusions

The results allow us to conclude that the HTST and UHT methods can allow for the physicochemical characteristics of the analyzed fruit nectars to be unchanged in terms of pH, TSS, and titratable acidity. All of the used methods allowed for the effective microbiological preservation of both types of nectars. HTST and UHT treatment reduced the turbidity of nectars after processing, most likely due to the thermal breakdown of macromolecular substances. The biggest color changes of nectars were observed after traditional pasteurization (PT), while the smallest changes were noted after HTST. Less intense changes in color and turbidity can be an added value for consumer acceptability. In the case of the total anthocyanin content. the HTST method provided the greatest retention of these substances immediately after the process in both nectars. In terms of total polyphenol content, no significant differences were observed between all of the preserved and raw nectars. Therefore, as a result of the HTST and UHT methods, we can obtain a product that lasts many months, losing fewer phenolic compounds, including anthocyanins, than conventional pasteurization. On the other hand, vitamin C is very sensitive to elevated temperatures, as each of the methods used caused its significant degradation in both nectars.

The varying effects of the thermal methods used on turbidity, antioxidant activity, or the content of bioactive components in nectars confirm the need for further research on the appropriate preservation parameters for products such as nectars and juices. Also, other quality distinctions important for consumer acceptance or health issues, such as the content of aromatic compounds or hydroxymethylfurfural, for example, should be taken into account during further evaluations of the impacts of preservation parameters. Considering our results, it is recommended to use the HTST and UHT methods under industrial conditions to process liquid fruit and vegetable products. It would be worthwhile to transfer the studied preservation parameters and analyze their effectiveness on an industrial scale. One limitation of our research was the storage time (only 6 months). It would be very valuable to extend this study by prolonging the shelf life of manufactured samples to 12 or even 24 months, like in industrial practice for pasteurized fruit products. The effect of the combined preservation methods involving HTST and UHT on the quality of liquid fruit and vegetable products should also be studied.

## Figures and Tables

**Table 1:** Impacts of processing methods and storage time on the pH, TA, and TSS of nectars.

Nectar	Month	pH				TA [% Citric Acid]				TSS [°Brix]			
		Raw	PT	HTST	UHT	Raw	PT	HTST	UHT	Raw	PT	HTST	UHT
SB	0	3.3 ± 0.0 ^A^	3.3 ± 0.0 ^Aa^	3.3 ± 0.0 ^Aa^	3.3 ± 0.0 ^Aa^	1.1 ± 0.0 ^A^	1.1 ± 0.0 ^Aa^	1.1 ± 0.0 ^Aa^	1.1 ± 0.0 ^Aa^	10.2 ± 0.0 ^A^	10.3 ± 0.0 ^Ba^	10.3 ± 0.1 ^Ba^	10.2 ± 0.1 ^Aa^
	6		3.3 ± 0.0 ^Aa^	3.3 ± 0.0 ^Aa^	3.3 ± 0.0 ^Aa^		1.1 ± 0.0 ^Aa^	1.1 ± 0.0 ^Aa^	1.1 ± 0.0 ^Aa^		10.3 ± 0.1 ^Ba^	10.2 ± 0.1 ^ABa^	10.1 ± 0.1 ^Aa^
SC	0	3.2 ± 0.0 ^A^	3.2 ± 0.1 ^Aa^	3.2 ± 0.2 ^Aa^	3.3 ± 0.1 ^Aa^	0.7 ± 0.0 ^A^	0.7 ± 0.0 ^Aa^	0.7 ± 0.0 ^Aa^	0.7 ± 0.0 ^Aa^	10.0 ± 0.1 ^AB^	10.1 ± 0.0 ^Ba^	9.9 ± 0.1 ^Aa^	10.0 ± 0.1 ^ABa^
	6		3.2 ± 0.0 ^Aa^	3.2 ± 0.0 ^Aa^	3.2 ± 0.0 ^Aa^		0.7 ± 0.0 ^Aa^	0.7 ± 0.0 ^Aa^	0.7 ± 0.0 ^Aa^		10.0 ± 0.0 ^Aa^	9.9 ± 0.1 ^Aa^	10.0 ± 0.1 ^Aa^

PT—traditional pasteurization, HTST—high-temperature short-time, UHT—ultra-high temperature, SB—strawberry–blackcurrant nectar, SC—strawberry–chokeberry nectar. The values in the same row with different capital letters are significantly different (*p* < 0.05) and concern changes between methods in specific months of storage. The values in the same column with different small letters are significantly different (*p* < 0.05) and concern changes during storage for specific methods.

**Table 2:** Impacts of processing methods and storage time on the NT of nectars.

Nectar	Month of Storage	NT [°NTU]			
		Raw	PT	HTST	UHT
SB	0	443 ± 9 ^BC^	356 ± 245 ^ABa^	490 ± 22 ^Ca^	282 ± 24 ^Aa^
	1	-	454 ± 219 ^Bab^	481 ± 18 ^Ba^	313 ± 70 ^Aab^
	2	-	397 ± 117 ^ABa^	483 ± 56 ^Ba^	295 ± 54 ^Aa^
	3	-	391 ± 267 ^ABa^	504 ± 97 ^Ba^	302 ± 10 ^Aa^
	4	-	494 ± 142 ^Bab^	511 ± 34 ^Ba^	369 ± 34 ^Abc^
	6	-	650 ± 11 ^Cb^	519 ± 62 ^Ba^	390 ± 64 ^Ac^
SC	0	85 ± 2 ^B^	71 ± 11 ^Ba^	70 ± 1 ^Ba^	29 ± 3 ^Aa^
	1	-	82 ± 8 ^Bab^	87 ± 2 ^Bb^	44 ± 3 ^Ab^
	2	-	90 ± 2 ^Bb^	100 ± 2 ^Cbc^	52 ± 5 ^Abc^
	3	-	86 ± 3 ^Bb^	109 ± 5 ^Ccd^	53 ± 5 ^Abc^
	4	-	86 ± 4 ^Bb^	115 ± 7 ^Cd^	60 ± 2 ^Ac^
	6	-	93 ± 14 ^Ab^	118 ± 9 ^Bd^	73 ± 3 ^Ad^

PT—traditional pasteurization, HTST—high-temperature short-time, UHT—ultra-high temperature, SB—strawberry–blackcurrant nectar, SC—strawberry–chokeberry nectar. The values in the same row with different capital letters are significantly different (*p* < 0.05) and concern changes between methods in specific months of storage. The values in the same column with different small letters are significantly different (*p* < 0.05) and concern changes during storage for specific methods.

**Table 3:** Impact of processing methods and storage time on the color parameters of nectars.

Nectar Parameters		Preservation Method	Raw Sample	After Preservation 0	Storage Time [Month]				
					1	2	3	4	6
SB	L*	PT	58.7 ± 0.3 ^A^	59.2 ± 0.3 ^Ca^	60.3 ± 0.5 ^Bab^	61.2 ± 0.3 ^Cb^	61.1 ± 0.3 ^Bb^	62.8 ± 0.5 ^ABc^	63.4 ± 0.7 ^Ac^
		HTST		58.0 ± 0.2 ^Ba^	57.9 ± 0.7 ^Aa^	59.6 ± 0.1 ^Ab^	59.6 ± 0.1 ^Ab^	61.8 ± 0.2 ^Ac^	63.2 ± 0.3 ^Ad^
		UHT		58.7 ± 0.2 ^Ca^	59.1 ± 1.1 ^ABa^	60.7 ± 0.1 ^Bb^	61.7 ± 0.2 ^Cb^	63.7 ± 0.5 ^Bc^	65.8 ± 0.7 ^Bd^
	a*	PT	67.5 ± 0.1 ^B^	66.5 ± 0.2 ^Ad^	63.5 ± 1.5 ^Ac^	63.0 ± 0.3 ^Ac^	62.0 ± 0.6 ^Abc^	60.8 ± 0.4 ^Bab^	59.2 ± 0.9 ^Ba^
		HTST		67.7 ± 0.1 ^Bf^	66.5 ± 0.5 ^Be^	65.1 ± 0.1 ^Cd^	64.0 ± 0.2 ^Bc^	62.5 ± 0.2 ^Cb^	59.8 ± 0.3 ^Ba^
		UHT		66.8 ± 0.1 ^Ae^	65.0 ± 0.5 ^ABd^	63.6 ± 0.1 ^Bd^	61.7 ± 0.4 ^Ac^	59.7 ± 0.4 ^Ab^	56.1 ± 1.4 ^Aa^
	b*	PT	37.8 ± 0.2 ^D^	32.7 ± 0.5 ^Ae^	28.4 ± 0.8 ^Ad^	26.2 ± 0.5 ^Ac^	24.0 ± 0.8 ^Ab^	23.3 ± 0.7 ^ABab^	21.8 ± 0.4 ^Ba^
		HTST		36.5 ± 0.5 ^Cf^	34.1 ± 1.6 ^Be^	30.1 ± 0.3 ^Cd^	27.6 ± 0.2 ^Bc^	25.1 ± 0.4 ^Bb^	22.0 ± 0.3 ^Ba^
		UHT		34.5 ± 0.4 ^Be^	30.6 ± 1.7 ^ABd^	27.2 ± 0.1 ^Bc^	23.9 ± 0.5 ^Ab^	22.0 ± 1.2 ^Ab^	19.1 ± 0.7 ^Aa^
SC	L*	PT	75.9 ± 0.1 ^B^	77.5 ± 0.4 ^Dab^	76.9 ± 1.3 ^Aa^	79.0 ± 0.3 ^Cbc^	79.0 ± 0.5 ^Babc^	79.2 ± 0.2 ^Bbc^	80.7 ± 1.2 ^Ac^
		HTST		75.4 ± 0.0 ^Aa^	77.9 ± 0.6 ^Ab^	76.2 ± 0.2 ^Aa^	77.2 ± 0.1 ^Ab^	77.9 ± 0.3 ^Ab^	79.1 ± 0.2 ^Ac^
		UHT		76.7 ± 0.0 ^Ca^	77.6 ± 0.8 ^Ab^	77.9 ± 0.1 ^Bb^	87.9 ± 0.0 ^Bc^	79.6 ± 0.0 ^Bc^	80.9 ± 0.2 ^Ad^
	a*	PT	52.3 ± 0.1 ^C^	48.7 ± 0.6 ^Ac^	47.5 ± 1.9 ^Ac^	43.7 ± 0.6 ^Ab^	43.6 ± 1.0 ^Ab^	43.1 ± 0.3 ^Aab^	39.7 ± 2.2 ^Aa^
		HTST		52.8 ± 0.1 ^Ce^	47.1 ± 0.9 ^Abc^	49.1 ± 0.1 ^Cd^	47.5 ± 0.0 ^Bc^	46.2 ± 0.5 ^Bb^	43.3 ± 0.3 ^Ba^
		UHT		50.2 ± 0.1 ^Bf^	47.4 ± 0.6 ^Ae^	45.9 ± 0.1 ^Bd^	43.9 ± 0.1 ^Ac^	42.5 ± 0.1 ^Ab^	39.5 ± 0.3 ^Aa^
	b*	PT	22.2 ± 0.1 ^C^	19.0 ± 0.5 ^Ac^	18.7 ± 1.4 ^Ac^	16.4 ± 0.3 ^Ab^	16.1 ± 0.5 ^Aab^	15.9 ± 0.1 ^Aab^	14.3 ± 0.9 ^Aa^
		HTST		22.7 ± 0.1 ^Ce^	18.6 ± 0.6 ^Abc^	20.4 ± 0.2 ^Cd^	18.9 ± 0.2 ^Cc^	18.0 ± 0.4 ^Bb^	16.1 ± 0.2 ^Ba^
		UHT		21.0 ± 0.1 ^Bf^	19.0 ± 0.5 ^Ae^	18.3 ± 0.1 ^Bd^	17.0 ± 0.1 ^Bc^	16.2 ± 0.1 ^Ab^	14.8 ± 0.1 ^Aa^

PT—traditional pasteurization, HTST—high temperature short time, UHT—ultra-high temperature, SB—strawberry–blackcurrant nectar, SC—strawberry–chokeberry nectar. The values in the same column with different capital letters are significantly different (*p* < 0.05) and concern changes between methods in specific months of storage. The values in the same row with different small letters are significantly different (*p* < 0.05) and concern changes during storage for specific methods.

**Table 4:** Impact of processing methods and storage time on the bioactive substance content in nectars.

Nectar Parameters		Preservation Method	Raw Sample	After Preservation 0	Storage Time [Month]				
					1	2	3	4	6
SB	Vitamin C [mg/100 mL]	PT	31.8 ± 0.3 ^C^	24.0 ± 0.9 ^Ab^	12.5 ± 5.3 ^Ba^	9.1 ± 5.4 ^Ba^	9.8 ± 1.6 ^Ba^	11.6 ± 2.5 ^Ba^	11.6 ± 3.3 ^Ba^
		HTST		25.3 ± 1.3 ^ABc^	6.1 ± 2.1 ^Ab^	2.7 ± 0.3 ^Aa^	1.3 ± 0.9 ^Aa^	1.0 ± 0.1 ^Aa^	1.0 ± 0.3 ^Aa^
		UHT		27.1 ± 0.7 ^Bc^	7.0 ± 1.4 ^Ab^	2.0 ± 0.6 ^Aa^	1.1 ± 0.6 ^Aa^	1.1 ± 0.4 ^Aa^	0.8 ± 0.2 ^Aa^
	TPC [mg GAE/ 100 mL]	PT	48.7 ± 1.9 ^A^	47.0 ± 0.6 ^Aab^	46.4 ± 0.7 ^Aa^	47.9 ± 0.6 ^Bab^	47.6 ± 0.0 ^Cab^	46.6 ± 0.0 ^Ca^	47.3 ± 0.5 ^Bab^
		HTST		47.8 ± 1.3 ^Ac^	44.8 ± 0.9 ^Ab^	45.1 ± 0.5 ^Ab^	43.3 ± 0.2 ^Aab^	42.2 ± 0.8 ^Aa^	44.9 ± 0.6 ^Ab^
		UHT		46.7 ± 2.7 ^Aab^	45.7 ± 1.1 ^Aab^	47.2 ± 0.3 ^Bb^	45.7 ± 0.2 ^Bab^	44.1 ± 0.8 ^Ba^	44.5 ± 1.2 ^Aa^
	TAC [mg/100 mL]	PT	36.7 ± 0.2 ^D^	30.7 ± 0.3 ^Ae^	26.5 ± 0.5 ^Ad^	23.6 ± 0.5 ^Ac^	21.6 ± 0.9 ^Ab^	21.1 ± 0.7 ^Bb^	18.1 ± 1.1 ^Ba^
		HTST		35.6 ± 0.2 ^Cf^	31.3 ± 0.6 ^Be^	27.6 ± 0.1 ^Bd^	25.4 ± 0.4 ^Bc^	23.3 ± 0.3 ^Cb^	19.0 ± 0.3 ^Ba^
		UHT		32.2 ± 0.0 ^Bf^	27.1 ± 0.2 ^Ae^	23.4 ± 0.2 ^Ad^	21.0 ± 0.3 ^Ac^	19.3 ± 0.5 ^Ab^	15.5 ± 1.0 ^Aa^
SC	Vitamin C [mg/100 mL]	PT	5.9 ± 0.3 ^C^	1.4 ± 0.2 ^Ab^	0.6 ± 0.0 ^Aa^	0.6 ± 0.1 ^Aa^	0.6 ± 0.0 ^Aa^	0.7 ± 0.0 ^Aa^	0.7 ± 0.^ABa^
		HTST		1.3 ± 0.1 ^Ac^	0.7 ± 0.0 ^Bab^	0.6 ± 0.1 ^Ba^	0.6 ± 0.0 ^Ba^	0.7 ± 0.0 ^Bab^	0.7 ± 0.1 ^Bb^
		UHT		1.9 ± 0.1 ^Bd^	0.6 ± 0.0 ^Aab^	0.6 ± 0.0 ^Aa^	0.6 ± 0.0 ^Aa^	0.7 ± 0.0 ^Abc^	0.7 ± 0.0 ^Ac^
	TPC [mg GAE/ 100 mL]	PT	63.5 ± 2.3 ^A^	65.9 ± 0.5 ^Ae^	36.6 ± 1.1 ^Ab^	38.5 ± 0.3 ^Cc^	38.5 ± 0.6 ^Ac^	40.5 ± 0.6 ^Cd^	28.4 ± 0.5 ^Ba^
		HTST		64.6 ± 1.8 ^Ac^	35.1 ± 1.0 ^Ab^	37.1 ± 0.2 ^Bb^	37.5 ± 0.6 ^Ab^	37.1 ± 0.3 ^Ab^	26.7 ± 0.2 ^Aa^
		UHT		65.6 ± 2.2 ^Ad^	36.3 ± 0.4 ^Abc^	35.5 ± 0.3 ^Ab^	38.7 ± 0.2 ^Ac^	38.5 ± 0.2 ^Bc^	27.4 ± 0.7 ^ABa^
	TAC [mg/100 mL]	PT	19.0 ± 0.1 ^D^	15.2 ± 0.2 ^Ad^	13.3 ± 0.2 ^Ac^	11.9 ± 0.4 ^Ab^	11.3 ± 0.3 ^Ab^	11.5 ± 0.3 ^Ab^	9.1 ± 1.0 ^Aa^
		HTST		18.3 ± 0.0 ^Cf^	16.4 ± 0.1 ^Ce^	14.8 ± 0.0 ^Cd^	13.9 ± 0.1 ^Bc^	13.3 ± 0.2 ^Bb^	11.1 ± 0.2 ^Ba^
		UHT		15.8 ± 0.1 ^Be^	13.9 ± 0.1 ^Bd^	12.6 ± 0.1 ^Bc^	11.5 ± 0.1 ^Ab^	11.4 ± 0.4 ^Ab^	9.2 ± 0.1 ^Aa^

PT—traditional pasteurization, HTST—high temperature short time, UHT—ultra-high temperature, SB—strawberry–blackcurrant nectar, SC—strawberry–chokeberry nectar, TPC—total phenolic content, TAC—total anthocyanins. The values in the same column with different capital letters are significantly different (p < 0.05) and concern changes between methods in specific months of storage. The values in the same row with different small letters are significantly different (p < 0.05) and concern changes during storage for specific methods.

**Table 5:** Effects of preservation methods and storage time on microbiological quality [CFU/ 1 mL] of nectars.

Nectar	Microbe	Preservation Method	Raw Sample	After Preservation 0	Storage Time [Month]				
					1	2	3	4	6
SB	MAMs	PT	2.5 × 10^2 C^ ± 7 × 10^1^	1.7 × 10^1 Ab^ ± 6 × 10^0^	3 × 10^0 Aa^ ± 6 × 10^0^	n.d.	n.d.	n.d.	n.d.
		HTST		2.0 × 10^2 Ca^ ± 4 × 10^1^	1.7 × 10^1 Bb^ ± 1.5 × 10^1^	n.d.	n.d.	n.d.	4.0 × 10^1 b^ ± 2.0 × 10^1^
		UHT		5.3 × 10^1 B^ ± 3.5 × 10^1^	n.d.	n.d.	n.d.	n.d.	n.d.
	LAB	PT	4.3 × 10^1^ ± 1.2 × 10^1^	n.d.	n.d.	n.d.	n.d.	n.d.	n.d.
		HTST		n.d.	n.d.	n.d.	n.d.	n.d.	n.d.
		UHT		n.d.	n.d.	n.d.	n.d.	n.d.	n.d.
	EFB	PT	n.d.	n.d.	n.d.	n.d.	n.d.	n.d.	n.d.
		HTST		n.d.	n.d.	n.d.	n.d.	n.d.	n.d.
		UHT		n.d.	n.d.	n.d.	n.d.	n.d.	n.d.
	AAB	PT	3.1 × 10^2 B^ ± 1.5 × 10^2^	2.5 × 10^1 Aa^ ± 9 × 10^0^	n.d.	n.d.	n.d.	n.d.	3.0 × 10^4 Bb^ ± 2 × 10^3^
		HTST		2.5 × 10^2 Ba^ ± 1.1 × 10^2^	n.d.	n.d.	n.d.	n.d.	4.2 × 10^4 Bb^ ± 9 × 10^3^
		UHT		6.2 × 10^1 Aa^ ± 1.0 × 10^1^	n.d.	n.d.	n.d.	n.d.	3.0 × 10^3 Ab^ ± 2 × 10^3^
	EB	PT	n.d.	n.d.	n.d.	n.d.	n.d.	n.d.	n.d.
		HTST		n.d.	n.d.	n.d.	n.d.	n.d.	n.d.
		UHT		n.d.	n.d.	n.d.	n.d.	n.d.	n.d.
	Y&M	PT	2.9 × 10^2^ ± 7 × 10^1^	n.d.	n.d.	n.d.	n.d.	n.d.	n.d.
		HTST		n.d.	n.d.	n.d.	n.d.	n.d.	n.d.
		UHT		n.d.	n.d.	n.d.	n.d.	n.d.	n.d.
SC	MAMs	PT	4.2 × 10^2 B^ ± 8 × 10^1^	n.d.	n.d.	n.d.	n.d.	n.d.	n.d.
		HTST		7 × 10^0 A^ ± 1.2 × 10^1^	n.d.	n.d.	n.d.	n.d.	n.d.
		UHT		3 × 10^0 A^ ± 6 × 10^0^	n.d.	n.d.	n.d.	n.d.	n.d.
	LAB	PT	1.5 × 10^1^ ± 5 × 10^0^	n.d.	n.d.	n.d.	n.d.	n.d.	n.d.
		HTST		n.d.	n.d.	n.d.	n.d.	n.d.	n.d.
		UHT		n.d.	n.d.	n.d.	n.d.	n.d.	n.d.
	EFB	PT	n.d.	n.d.	n.d.	n.d.	n.d.	n.d.	n.d.
		HTST		n.d.	n.d.	n.d.	n.d.	n.d.	n.d.
		UHT		n.d.	n.d.	n.d.	n.d.	n.d.	n.d.
	AAB	PT	2.9 × 10^2 B^ ± 7 × 10^1^	3.1 × 10^1 Aa^ ± 1.1 × 10^1^	n.d.	n.d.	n.d.	n.d.	3.7 × 10^3 Bb^ ± 5 × 10^2^
		HTST		2.9 × 10^1 Aa^ ± 1.5 × 10^1^	n.d.	n.d.	n.d.	n.d.	2.1 × 10^2 Ab^ ± 1.1 × 10^2^
		UHT		7.0 × 10^1 Aa^ ± 9 × 10^0^	n.d.	n.d.	n.d.	n.d.	4.4 × 10^2 ab^ ± 5 × 10^1^
	EB	PT	n.d.	n.d.	n.d.	n.d.	n.d.	n.d.	n.d.
		HTST		n.d.	n.d.	n.d.	n.d.	n.d.	n.d.
		UHT		n.d.	n.d.	n.d.	n.d.	n.d.	n.d.
	Y&M	PT	2.3 × 10^2^ ± 5 × 10^1^	n.d.	n.d.	n.d.	n.d.	n.d.	n.d.
		HTST		n.d.	n.d.	n.d.	n.d.	n.d.	n.d.
		UHT		n.d.	n.d.	n.d.	n.d.	n.d.	n.d.

MAMs—mesophilic aerobic microorganisms, LAB—lactic acid bacteria, EFB—endospore-forming bacteria, AAB—acetic acid bacteria, EB—*Enterobacteriaceae*, Y&M—yeast and mold; PT—traditional pasteurization, HTST—high temperature short time, UHT—ultra-high temperature; SB—strawberry–blackcurrant nectar, SC—strawberry–chokeberry nectar. The values in the same column with different capital letters are significantly different (*p* < 0.05) and concern changes between methods in specific months of storage. The values in the same row with different small letters are significantly different (*p* < 0.05) and concern changes during storage for the specific method.

## Data Availability

The original contributions presented in this study are included in the article. Further inquiries can be directed to the corresponding authors.

## Appendix A

**Table A1:** Effect of preservation methods and storage time on individual anthocyanin content [mg/100 mL] in nectar.

Nectar	Anthocyanin	Preservation Method	Raw Sample	After Preservation 0	Storage Time [Months]				
					1	2	3	4	6
SB	Dp-3-*O*-glu	PT	5.0 ± 0.1 ^C^	4.01 ± 0.1 ^Ae^	3.2 ± 0.1 ^Ad^	2.9 ± 0.0 ^Ac^	2.7 ± 0.1 ^Abc^	2.6 ± 0.1 ^Bb^	2.2 ± 0.1 ^Ba^
		HTST		5.0 ± 0.0 ^Cf^	4.0 ± 0.1 ^Be^	3.6 ± 0.0 ^Bd^	3.3 ± 0.1 ^Bc^	3.1 ± 0.0 ^Cb^	2.5 ± 0.0 ^Ca^
		UHT		4.3 ± 0.1 ^Bf^	3.3 ± 0.1 ^Ae^	2.9 ± 0.0 ^Ad^	2.6 ± 0.1 ^Ac^	2.4 ± 0.1 ^Ab^	1.9 ± 0.1 ^Aa^
	Dp-3-*O*-rut	PT	14.3 ± 0.1 ^D^	11.5 ± 0.1 ^Ae^	10.0 ± 0.1 ^Ad^	9.0 ± 0.2 ^Ac^	8.3 ± 0.3 ^Abc^	8.0 ± 0.3 ^Bb^	7.0 ± 0.4 ^Ba^
		HTST		13.7 ± 0.1 ^Cf^	12.0 ± 0.2 ^Be^	10.8 ± 0.1 ^Bd^	9.8 ± 0.2 ^Bc^	9.0 ± 0.1 ^Cb^	7.4 ± 0.1 ^Ba^
		UHT		12.2 ± 0.0 ^Bf^	10.3 ± 0.1 ^Ae^	9.0 ± 0.1 ^Ad^	8.1 ± 0.1 ^Ac^	7.4 ± 0.2 ^Ab^	6.0 ± 0.4 ^Aa^
	Cy-3-*O*-glu	PT	1.8 ± 0.0 ^B^	1.6 ± 0.1 ^Ae^	1.4 ± 0.0 ^Ad^	1.3 ± 0.0 ^Bbc^	1.2 ± 0.0 ^Ab^	1.4 ± 0.0 ^Acd^	1.0 ± 0.1 ^Ba^
		HTST		1.8 ± 0.0 ^Bd^	1.7 ± 0.0 ^Bc^	1.4 ± 0.1 ^Cb^	1.4 ± 0.0 ^Bb^	1.6 ± 0.0 ^Bc^	1.1 ± 0.0 ^Ba^
		UHT		1.6 ± 0.0 ^Ae^	1.5 ± 0.0 ^Ad^	1.2 ± 0.0 ^Ab^	1.2 ± 0.0 ^Ab^	1.3 ± 0.1 ^Ac^	0.9 ± 0.1 ^Aa^
	Cy-3-*O*-rut	PT	7.9 ± 0.1 ^D^	6.6 ± 0.1 ^Ae^	5.8 ± 0.1 ^Ad^	5.2 ± 0.1 ^Ac^	4.7 ± 0.2 ^Abc^	4.6 ± 0.2 ^Bb^	4.0 ± 0.2 ^Ba^
		HTST		7.4 ± 0.1 ^Cf^	6.7 ± 0.1 ^Be^	5.9 ± 0.0 ^Bd^	5.4 ± 0.1 ^Bc^	4.9 ± 0.1 ^Bb^	4.1 ± 0.1 ^Ba^
		UHT		6.9 ± 0.0 ^Bf^	5.9 ± 0.1 ^Ae^	5.1 ± 0.0 ^Ad^	4.6 ± 0.1 ^Ac^	4.2 ± 0.1 ^Ab^	3.4 ± 0.2 ^Aa^
	Pg-3-*O*-glu	PT	7.5 ± 0.1 ^C^	6.7 ± 0.0 ^Ae^	5.9 ± 0.2 ^Ad^	5.1 ± 0.2 ^Ac^	4.5 ± 0.2 ^Ab^	4.3 ± 0.2 ^Bb^	3.7 ± 0.3 ^Ba^
		HTST		7.4 ± 0.1 ^Cf^	6.8 ± 0.1 ^Be^	5.7 ± 0.0 ^Bd^	5.1 ± 0.1 ^Bc^	4.6 ± 0.1 ^Bb^	3.7 ± 0.1 ^Ba^
		UHT		7.0 ± 0.0 ^Bf^	5.9 ± 0.1 ^Ae^	4.9 ± 0.0 ^Ad^	4.3 ± 0.1 ^Ac^	3.8 ± 0.1 ^Ab^	3.0 ± 0.2 ^Aa^
	Pg-3-*O*-ara	PT	0.3 ± 0.0 ^A^	0.3 ± 0.0 ^Ac^	0.2 ± 0.0 ^Aab^	0.2 ± 0.0 ^Aab^	0.2 ± 0.0 ^Abc^	0.2 ± 0.0 ^Aabc^	0.2 ± 0.0 ^Aa^
		HTST		0.3 ± 0.0 ^Ae^	0.2 ± 0.0 ^Ac^	0.2 ± 0.0 ^Ab^	0.3 ± 0.0 ^Ad^	0.2 ± 0.0 ^Ac^	0.2 ± 0.0 ^Aa^
		UHT		0.3 ± 0.0 ^Ad^	0.2 ± 0.0 ^Ac^	0.2 ± 0.0 ^Aa^	0.2 ± 0.0 ^Ac^	0.2 ± 0.0 ^Ab^	0.2 ± 0.0 ^Aa^
SC	Cy-3-*O*-gal	PT	9.4 ± 0.1 ^D^	7.3 ± 0.1 ^Ad^	6.2 ± 0.1 ^Ac^	5.6 ± 0.2 ^Abc^	5.3 ± 0.2 ^Ab^	5.3 ± 0.1 ^Ab^	4.2 ± 0.5 ^Aa^
		HTST		8.9 ± 0.0 ^Cf^	7.8 ± 0.1 ^Ce^	7.0 ± 0.0 ^Cd^	6.5 ± 0.1 ^Bc^	6.2 ± 0.1 ^Ab^	5.2 ± 0.1 ^Ba^
		UHT		7.6 ± 0.0 ^Bc^	6.6 ± 0.0 ^Bbc^	6.0 ± 0.0 ^Bb^	5.4 ± 0.1 ^Aab^	5.8 ± 1.1 ^Ab^	4.3 ± 0.1 ^Aa^
	Cy-3-*O*-glu	PT	0.5 ± 0.0 ^C^	0.4 ± 0.0 ^Abc^	0.4 ± 0.0 ^Ac^	0.4 ± 0.0 ^Ab^	0.4 ± 0.0 ^Ab^	0.6 ± 0.0 ^Bd^	0.3 ± 0.0 ^Aa^
		HTST		0.5 ± 0.0 ^BCc^	0.5 ± 0.0 ^Cc^	0.4 ± 0.0 ^Bb^	0.4 ± 0.0 ^Bb^	0.6 ± 0.0 ^ABd^	0.4 ± 0.0 ^Aa^
		UHT		0.5 ± 0.0 ^Bc^	0.5 ± 0.0 ^Bc^	0.4 ± 0.0 ^Ab^	0.4 ± 0.0 ^Ab^	0.6 ± 0.0 ^Ad^	0.3 ± 0.0 ^Aa^
	Cy-3-*O*-ara	PT	3.9 ± 0.0 ^C^	2.7 ± 0.0 ^Ad^	2.4 ± 0.1 ^Ac^	2.1 ± 0.1 ^Ab^	2.0 ± 0.1 ^Ab^	2.0 ± 0.1 ^Bb^	1.6 ± 0.2 ^Aa^
		HTST		3.7 ± 0.0 ^Bf^	3.2 ± 0.0 ^Be^	2.8 ± 0.0 ^Bd^	2.6 ± 0.0 ^Bc^	2.4 ± 0.0 ^Cb^	2.0 ± 0.0 ^Ba^
		UHT		2.8 ± 0.0 ^Ac^	2.5 ± 0.0 ^Abc^	2.2 ± 0.0 ^Aabc^	2.0 ± 0.0 ^Aab^	1.4 ± 0.7 ^Aa^	1.5 ± 0.0 ^Aa^
	Pg-3-*O*-glu	PT	4.5 ± 0.0 ^C^	4.3 ± 0.0 ^Ad^	3.8 ± 0.0 ^Ac^	3.4 ± 0.1 ^Ab^	3.2 ± 0.1 ^Ab^	3.2 ± 0.1 ^Ab^	2.6 ± 0.3 ^Aa^
		HTST		4.5 ± 0.0 ^Cf^	4.3 ± 0.0 ^Ce^	3.9 ± 0.0 ^Cd^	3.7 ± 0.0 ^Bc^	3.5 ± 0.0 ^Bb^	3.0 ± 0.1 ^Aa^
		UHT		4.4 ± 0.0 ^Bf^	3.9 ± 0.0 ^Be^	3.6 ± 0.1 ^Bd^	3.3 ± 0.0 ^Ac^	3.1 ± 0.0 ^Ab^	2.7 ± 0.0 ^Aa^
	Pg-3-*O*-ara	PT	0.2 ± 0.0 ^A^	0.2 ± 0.0 ^Ac^	0.2 ± 0.0 ^Ac^	0.2 ± 0.0 ^Abc^	0.2 ± 0.0 ^Abc^	0.2 ± 0.0 ^Abc^	0.2 ± 0.0 ^Aa^
		HTST		0.2 ± 0.0 ^Ac^	0.2 ± 0.0 ^Cd^	0.2 ± 0.0 ^Bc^	0.2 ± 0.0 ^Cc^	0.2 ± 0.0 ^Bb^	0.2 ± 0.0 ^Ba^
		UHT		0.2 ± 0.0 ^Ac^	0.2 ± 0.0 ^Bc^	0.2 ± 0.0 ^Ab^	0.2 ± 0.0 ^Bc^	0.2 ± 0.0 ^Ab^	0.2 ± 0.0 ^ABa^
	Cy-3-*O*-xyl	PT	0.5 ± 0.0 ^C^	0.4 ± 0.0 ^Ad^	0.3 ± 0.0 ^Ac^	0.3 ± 0.0 ^Aab^	0.3 ± 0.0 ^Abc^	0.3 ± 0.0 ^Abc^	0.2 ± 0.0 ^Aa^
		HTST		0.5 ± 0.0 ^Be^	0.4 ± 0.0 ^Bd^	0.4 ± 0.0 ^Bbc^	0.4 ± 0.0 ^Bc^	0.3 ± 0.0 ^Bb^	0.3 ± 0.0 ^Ba^
		UHT		0.4 ± 0.0 ^Ae^	0.3 ± 0.0 ^Ad^	0.3 ± 0.0 ^Abc^	0.3 ± 0.0 ^Ac^	0.2 ± 0.0 ^Ab^	0.2 ± 0.0 ^Aa^

Cy-3-*O*-ara—cyanidin-3-*O*-arabinoside, cy-3-*O*-gal—cyanidin-3-*O*-galactoside, cy-3-*O*-glu—cyanidin-3-*O*-glucoside, cy-3-*O*-rut—cyanidin-3-*O*-rutinoside, cy-3-*O*-xyl—cyanidin-3-*O*-xyloside, dp-3-*O*-glu—delphinidin-3-*O*-glucoside, dp-3-*O*-rut—delphinidin-3-*O*-rutinoside, pg-3-*O*-ara—pelargonidin-3-*O*-arabinoside, pg-3-O-glu—pelargonidin-3-O-glucoside; PT—traditional pasteurization, HTST—high temperature short time, UHT—ultra-high temperature; SB—strawberry–blackcurrant nectar, SC—strawberry–chokeberry nectar. The values in the same column with different capital letters are significantly different (*p* < 0.05) and concern changes between methods in specific months of storage. The values in the same row with different small letters are significantly different (*p* < 0.05) and concern changes during storage for specific methods.
